# Anxiolytic Potential of Selected Essential Oils and Their Impact on Motor Function

**DOI:** 10.3390/ijms27198786

**Published:** 2026-09-30

**Authors:** Alberto Souza Sá Filho, John Ihayi Ogbu, Ayobami J. Olusola, Saheed Olanrewaju Afolabi, Gustavo Rodrigues Pedrino, Marcos Luiz Ferreira-Neto, Vicente Aprigliano, Renê Oliveira do Couto, Lucas Barbosa Ribeiro de Carvalho, Leonardo Luiz Borges, Mirely Vitória Farias da Silva, Edeildo Ferreira da Silva-Júnior, Antônio Sérgio Nakao de Aguiar, James Oluwagbamigbe Fajemiroye

**Affiliations:** 1 Graduate Program in Pharmaceutical Sciences, Evangelical University of Goiás, Anápolis 75083-515, Brazil; doutor.alberto@outlook.com (A.S.S.F.); toninho.quimica@gmail.com (A.S.N.d.A.); 2 Institute of Biological Sciences, Federal University of Goiás, Goiânia 74690-900, Brazil; ogbu11john@gmail.com (J.I.O.); ayobamiolusola@discente.ufg.br (A.J.O.); pedrino@ufg.br (G.R.P.); 3 Department of Pharmacology and Therapeutics, University of Ilorin, Ilorin 240003, Nigeria; afolabi023@gmail.com; 4 Department of Physiology, Federal University of Uberlândia, Uberlândia 38405-320, Brazil; marcosneto@ufu.br; 5 Escuela de Ingeniería de Construcción y Transporte, Pontifícia Universidad Católica de Valparaíso, Avda Brasil 2147, Valparaíso 2362804, Chile; 6 Laboratory of Pharmaceutical Development (LADEF), Federal University of São João del-Rei, Divinópolis 35501-296, Brazil; rocouto@ufsj.edu.br; 7 School of Medical and Life Sciences, Pontifical Catholic University of Goiás, Goiânia 74605-010, Brazil; lubarica@gmail.com (L.B.R.d.C.); leonardoquimica@gmail.com (L.L.B.); 8 Laboratory for Research, Development & Innovation of Biodiversity Products, State University of Goiás, Anápolis 75132-903, Brazil; 9 Institute of Chemistry and Biotechnology, Federal University of Alagoas, AC Simões Campus, Maceió 57072-970, Brazil; mirely.silva@iqb.ufal.br (M.V.F.d.S.); edeildo.junior@iqb.ufal.br (E.F.d.S.-J.)

**Keywords:** anti-anxiety, natural products, essential oils, motor skills disorders

## Abstract

Plant essential oils (EOs) exhibit promising anxiolytic activity, but their application may be constrained by adverse motor effects arising from interactions with neural circuits controlling movement. Here, we investigated the anxiolytic-like effects and motor impairment liability of EOs from *Pimenta pseudocaryophyllus*, *Melissa officinalis*, *Matricaria chamomilla*, *Lippia alba*, *Salvia rosmarinus*, *Ocimum basilicum*, *Spiranthera odoratissima*, and *Cymbopogon citratus*, integrating computational target prediction with behavioral pharmacology. Prioritized EO constituents were subjected to target prediction, molecular docking, and 100-ns molecular dynamics simulations to identify molecular mechanisms potentially associated with motor impairment. Male and female Swiss mice received oral vehicle 10 mL/kg, EOs (10, 100, or 500 mg/kg) or diazepam (DZP 1 or 5 mg/kg), followed by the evaluation of anxiolytic-like behavior and motor coordination in the elevated plus maze and rotarod test, respectively. In silico analyses involving docking and molecular dynamics simulations of computationally prioritized EO constituents identified candidate targets within M1 and M2 receptors. All EOs exhibited anxiolytic-like activity at the intermediate dose. Motor impairment varied markedly among EOs. Integrating computational target prediction with behavioral assessment identified distinct motor liability profiles among EOs.

## 1. Introduction

Motor incoordination, a well-recognized adverse effect of conventional anxiolytics, often limits therapeutic utility and patient compliance [1]. Benzodiazepines, for example, exert their effects through positive allosteric modulation of GABAA receptors. This mechanism is intrinsically linked to dose-dependent impairments in motor coordination, ataxia, and increased fall risk in vulnerable elderly populations [2,3]. Similar liabilities are observed with other centrally acting agents, including barbiturates, some antidepressants, and anxiolytics, thereby reflecting a narrow separation between anxiolysis and motor incoordination [4]. These limitations underscore the need for anxiolytic screening to improve safety margins.

Essential oils (EOs) are complex mixtures of bioactive compounds (Table S1), many of which may contribute to their pharmacological effects. Accordingly, relaxant and sleep-promoting effects have been documented in traditional use and preclinical studies, with growing evidence from clinical studies [5,6]. Species such as *Melissa officinalis* and *Matricaria chamomilla* have long been used for anxiety and insomnia [7,8]. However, unlike single-target drugs, EOs exhibit multitarget activity across diverse CNS pathways, which may also contribute to adverse motor outcomes.

Despite their therapeutic promise, translating EOs into evidence-based anxiolytic interventions remains limited [9]. Their pharmacological effects are strongly dose- and composition-dependent: while lower doses may reduce anxiety, higher doses can induce sedation, motor impairment, or cardiovascular effects depending on their chemical profile [10]. In addition, their chemical complexity often complicates mechanistic attribution and raises the risk of off-target interactions, including with other CNS depressants. These challenges highlight the need for systematic approaches that integrate efficacy with safety profiling early in development.

Recent advances in predictive pharmacology and behavioral neuroscience now enable integrated evaluation of EOs’ bioactivity. In silico target prediction and molecular docking can generate mechanistic hypotheses, while standardized behavioral assays allow parallel assessment of anxiolytic-like effects and motor coordination. Together, these approaches provide a framework for defining dose–response relationships and safety margins and for prioritizing candidates with improved therapeutic profiles [11].

Here, we combine in silico target prediction and molecular docking with in vivo behavioral assays to evaluate EOs from *Pimenta pseudocaryophyllus* (Gomes) *Landrum*, *Melissa officinalis* L., *Matricaria chamomilla* L., *Lippia alba* (Mill.), *Salvia rosmarinus Spenn.*, *Spiranthera odoratissima A. St.-Hil.*, *Ocimum basilicum* L., and *Cymbopogon citratus* (DC.) Stapf. These EOs were selected based on preclinical evidence, as well as traditional and ethnopharmacological reports describing the use of their source plant species for promoting relaxation, alleviating anxiety-like symptoms, and modulating sleep. By integrating dose–response analyses of anxiolytic-like activity and motor performance, we aim to delineate safety boundaries and identify potential constituent-target interactions for further studies.

## 2. Results

### 2.1. Docking and Molecular Dynamics (MD) Simulations

The molecular docking analysis presented in Table S2 enables the comparison of the relative affinity of the compounds toward the muscarinic receptors M1 and M2, highlighting differences in interaction profiles between receptor subtypes. Both receptors were considered potentially involved in the essential oil’s activity. Physiologically, muscarinic receptors play distinct roles in the central nervous system. The M1 receptor couples to the Gq protein, promoting increased neuronal activity, whereas the M2 receptor couples to the Gi protein and is associated with reduced neuronal excitability. Overall, the results indicate a trend of differential ligand affinity. Chavibetol, β-caryophyllene, neral, and geraniol (present in all EOs) exhibited higher predicted affinity for the M1 receptor, whereas linalool and eucalyptol (present in all EOs) showed greater affinity for the M2 receptor. These differences suggest variations in structural complementarity between the ligands and the receptor binding sites, reflecting potential distinctions in the topology of the binding cavities between M1 and M2. In this context, the higher affinity observed for each receptor subtype may indicate differences in cholinergic modulation.

**Figure 1:**
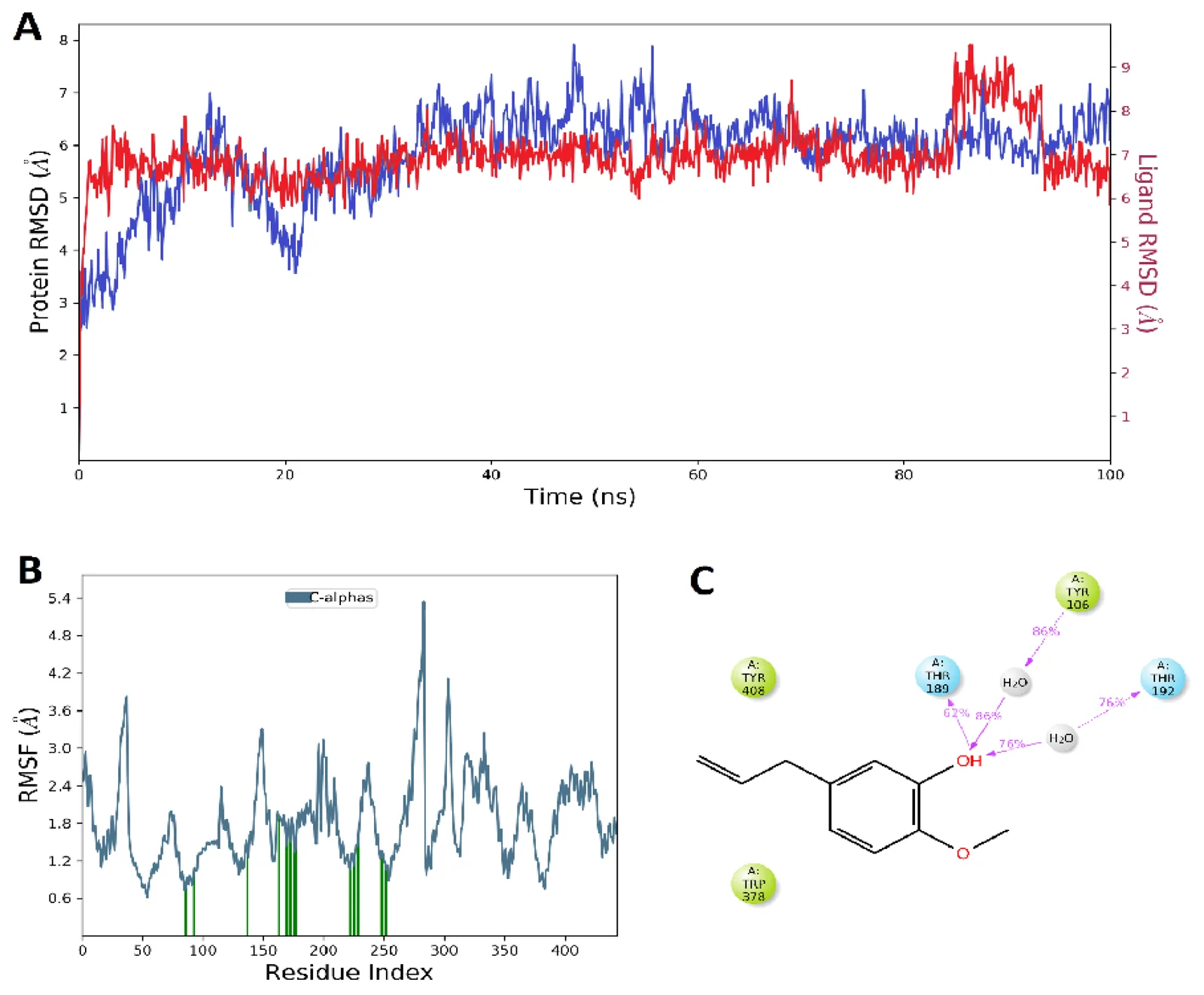
Molecular dynamics simulations for the muscarinic M1 receptor in complex with chavibetol within 100 ns for RMSD (**A**), RMSF (**B**), and interaction (**C**) plots. In (**A**), protein and ligand are represented by blue and red lines, respectively. In (**B**), the vertical green lines represent the ligand contacts within the simulation. In (**C**), the %values mean the frequency of binding for each amino acid residue or water molecule during the simulation.

MD simulations provided relevant insights into the conformational stability of the complex formed between the muscarinic M1 receptor and chavibetol. Overall, the system reached equilibrium after an initial period of structural accommodation, with the ligand remaining within the binding cavity throughout the simulation. The RMSD plot (Figure 1A) indicates a relatively stable system, albeit with structural changes along the trajectory. The M1 backbone starts near 3.0 Å and rapidly increases to the range of 5.5–7.0 Å (RMSD_average_: 6.3 Å), where it remains for most of the simulation. This behavior reflects initial structural relaxation followed by stabilization with moderate fluctuations, suggesting global conformational flexibility consistent with proteins in the G protein-coupled receptor (GPCR) family [12]. Chavibetol exhibited RMSD values predominantly between 5.0 and 6.5 Å (RMSD_average_: 6.0 Å) relative to the protein, indicating its retention within the binding site. However, a more pronounced increase is observed between approximately 80 and 95 ns, reaching values close to 8–9 Å, suggesting conformational rearrangements within the binding cavity. Following this period, the RMSD returns to lower values, indicating re-stabilization of the system without evidence of ligand dissociation.

The RMSD results indicate that, despite the receptor’s structural flexibility, chavibetol maintains a stable interaction throughout the simulation, suggesting good conformational adaptation within the binding cavity. The RMSF plot (Figure 1B) reveals that most residues fluctuate between ~1.0 and 2.5 Å, indicating overall structural stability of the protein C_α_. However, higher peaks are observed, particularly in the region spanning residues ~270–300, reaching values above 4.0 Å, which characterizes more flexible regions. In the muscarinic M1 receptor, this interval corresponds to the intracellular portion of transmembrane helix 6 (TM6) and the third intracellular loop (ICL3), a region known for its high conformational mobility and involvement in G protein coupling. In molecular dynamics simulations of G protein-coupled receptors (GPCRs), this region is frequently described as partially disordered and solvent-exposed [13]. The residues in contact with the ligand are mainly concentrated in the regions ~90–100, ~150–180, and ~230–250. In these regions, RMSF values remain low to moderate (~1.0–2.0 Å), indicating that the binding site remains structurally stable throughout the simulation. This behavior supports the sustained presence of chavibetol within the binding cavity, despite the overall flexibility of M1. Molecular interaction analysis showed that chavibetol maintains a consistent set of contacts with residues in the orthosteric site of the muscarinic M1 receptor throughout the simulation, contributing to its stability. Among the most frequently interacting residues, TYR106, THR189, and THR192 stand out, with TYR106 as the primary contributor to ligand anchoring (Figure 1C).

**Figure 2:**
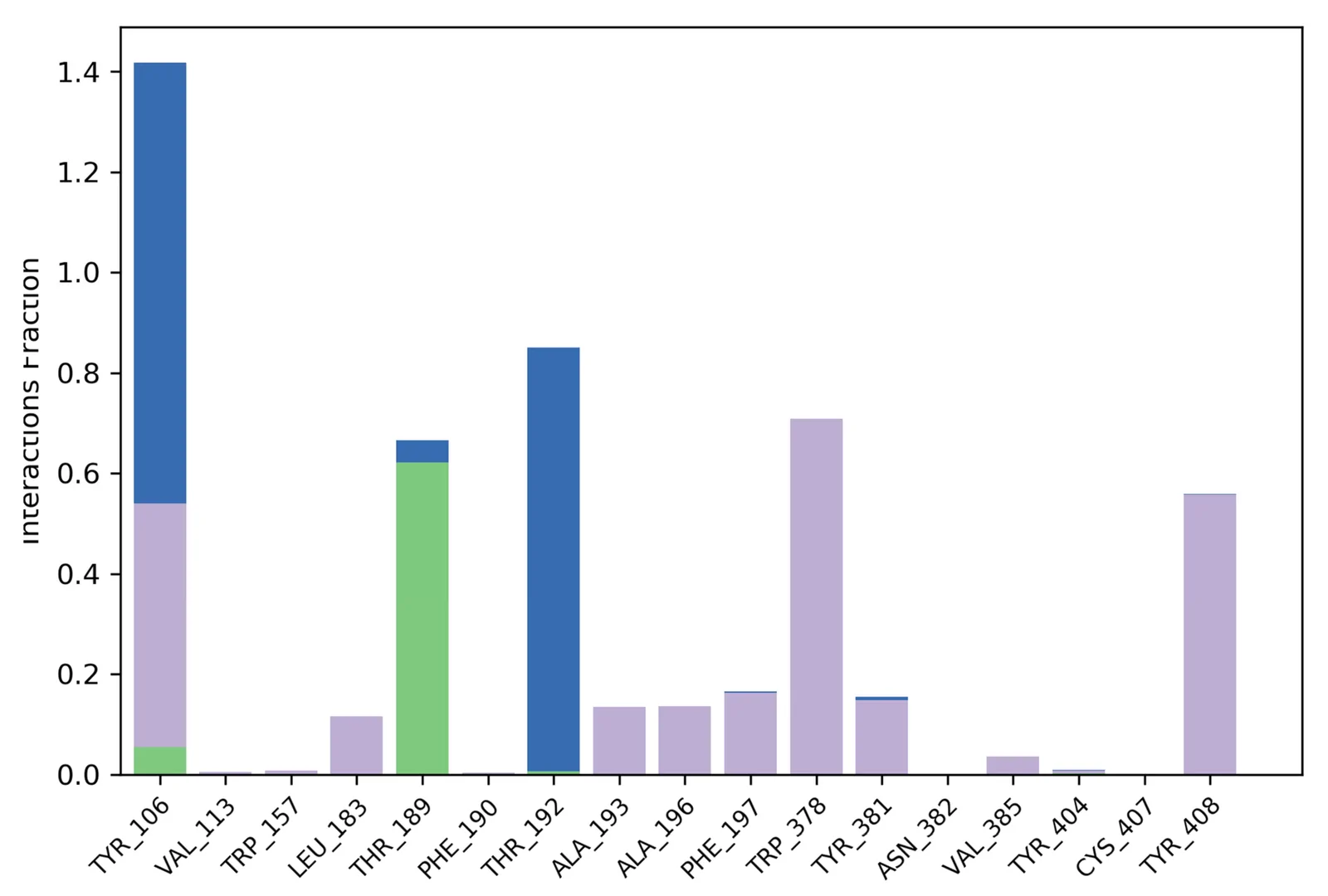
Interaction fraction between the muscarinic M1 receptor and chavibetol during the 100 ns molecular dynamics simulation. Hydrophobic contacts, water-bridge interactions, and H-bonding interactions are represented by purple, blue, and green bars, respectively.

These interactions are predominantly polar, including hydrogen bonds that are often water-mediated, and exhibit high occupancy rates (above ~60–80%), indicating a persistent interaction profile. In addition, aromatic residues such as TRP378, TYR381, and TYR408 (Figure 2) participate in hydrophobic and π-type interactions, contributing to the accommodation of chavibetol within an aromatic-rich binding pocket. This structural pattern is characteristic of the orthosteric site of muscarinic receptors, as described in the literature [14,15]. Moreover, the balance between hydrophobic and polar interactions has been widely described as a key determinant of molecular recognition in muscarinic receptors [16,17]. Overall, the interaction profile indicates that chavibetol fits within an aromatic- and polar-rich binding site, balancing hydrophobic contacts with hydrogen bonding. This behavior is consistent with the RMSF data, which indicates low mobility of residues directly involved in ligand binding.

Thus, the results support that chavibetol exhibits a stable binding mode compatible with the structural characteristics of the muscarinic M1 receptor, maintaining persistent interactions throughout the molecular dynamics simulation. However, experimental validation is required to determine its functional profile as an agonist or antagonist.

### 2.2. Elevated Plus Maze

**Figure 3:**
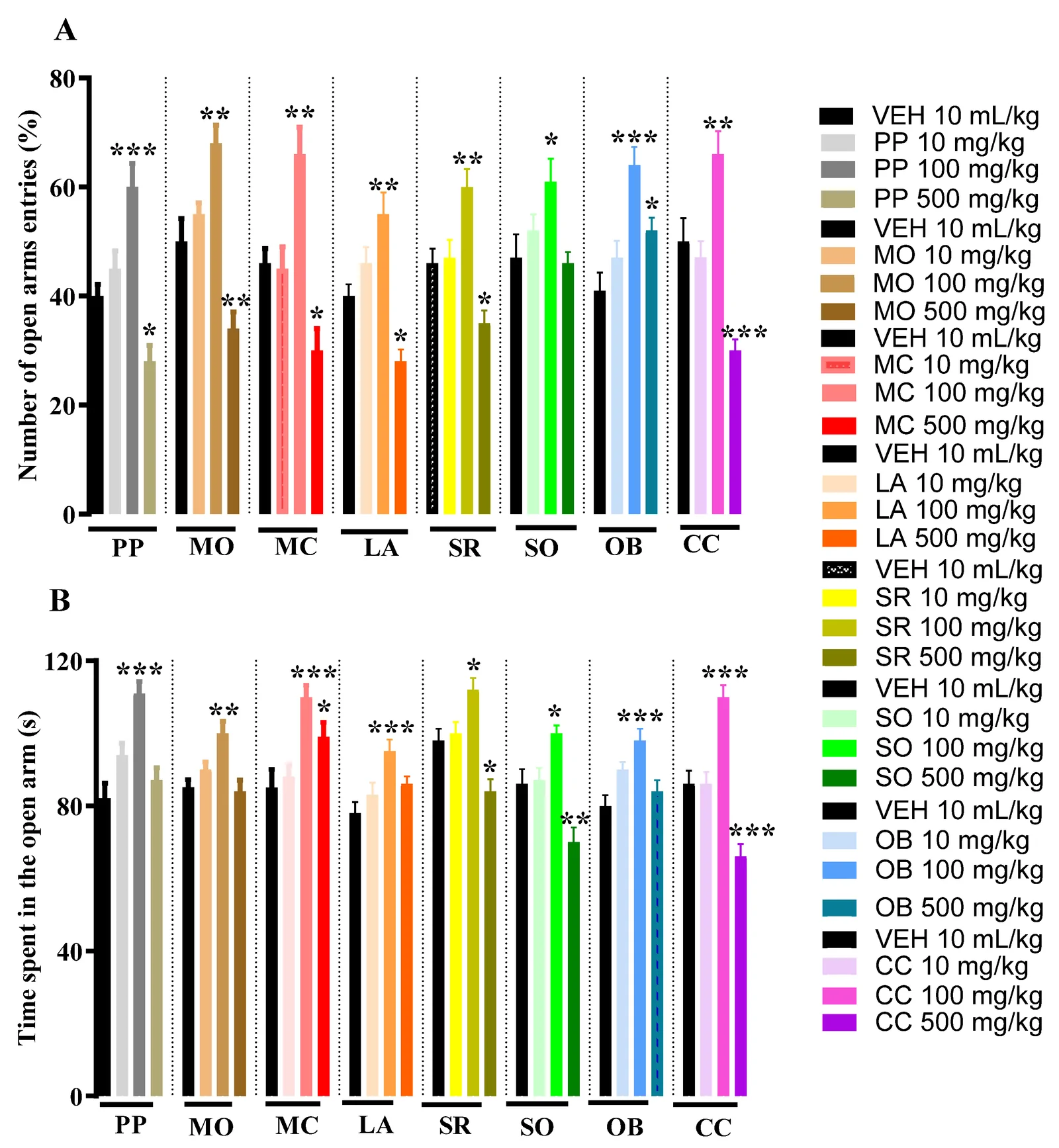
Effect of vehicle (VEH), EOs of *Pimenta pseudocaryophyllus* (PP), *Melissa officinalis* (MO), *Matricaria chamomilla* (MC), *Lippia alba* (LA), *Salvia rosmarinus* (SR), *Spiranthera odoratissima* (SO), *Ocimum basilicum* (OB), and *Cymbopogon citratus* (CC) in the elevated plus maze test. The experiments were carried out in mice 60 min after oral treatment with VEH 10 mL/kg, PP, MO, MC, LA, SR, SO, OB, and CC at doses of 10, 100, and 500 mg/kg. The parameters evaluated were the number of open arm entries (**A**) and time spent in the open arms (**B**). Each column represents the mean ± S.E.M. (*n* = 10 Swiss mice per group; 5 males and 5 females). Statistical analysis was performed by one-way ANOVA followed by Dunnett’s post hoc test for multiple comparisons. * *p* < 0.05, ** *p* < 0.01, and *** *p* < 0.001 other treatment groups vs. vehicle treated group.

In the EPM, statistically significant differences were observed among experimental groups (Detailed experimental data with diazepam are in Table S3). In Figure 3A, a significant increase in the number of open arm entries was observed in the group treated at the dose of 100 mg/kg of EOs [*Pimenta pseudocaryophyllus*, PP; *Melissa officinalis*, MO; *Matricaria chamomilla*, MC; *Lippia alba*, LA; *Salvia rosmarinus*, SR; *Ocimum basilicum*, OB; *Spiranthera odoratissima*, SO; and *Cymbopogon citratus*, CC; *p* < 0.05] and a significant reduction in the number of open arm entries in the group treated at the dose of 500 mg/kg of EOs [PP: F_(3,36)_ = 16.12; MO: F_(3,36)_ = 18.46; MC: F_(3,36)_ = 13.58; LA: F_(3,36)_ = 14.79; SR: F_(3,36)_ = 11.91; SO: F_(3,36)_ = 3.77; OB: F_(3,36)_ = 10.12; and CC: F_(3,36)_ = 17.44; *p* < 0.05, Figure 3A]. As shown in Figure 3B, a significant increase in the time spent in the open arms of EPM in the group treated at the dose of 100 mg/kg and a significant reduction in the time spent in the open arms of EPM in the group treated at the dose of 500 mg/kg (PP: F_(3,36)_ = 12.44; MO: F_(3,36)_ = 7.12; MC: F_(3,36)_ = 8.21, LA: F_(3,36)_ = 5.84; SR: F_(3,36)_ = 12.30; SO: F_(3,36)_ = 12.27; OB: F_(3,36)_ = 7.29; and CC: F_(3,36)_ = 27.30).

### 2.3. Rotarod Test

**Figure 4:**
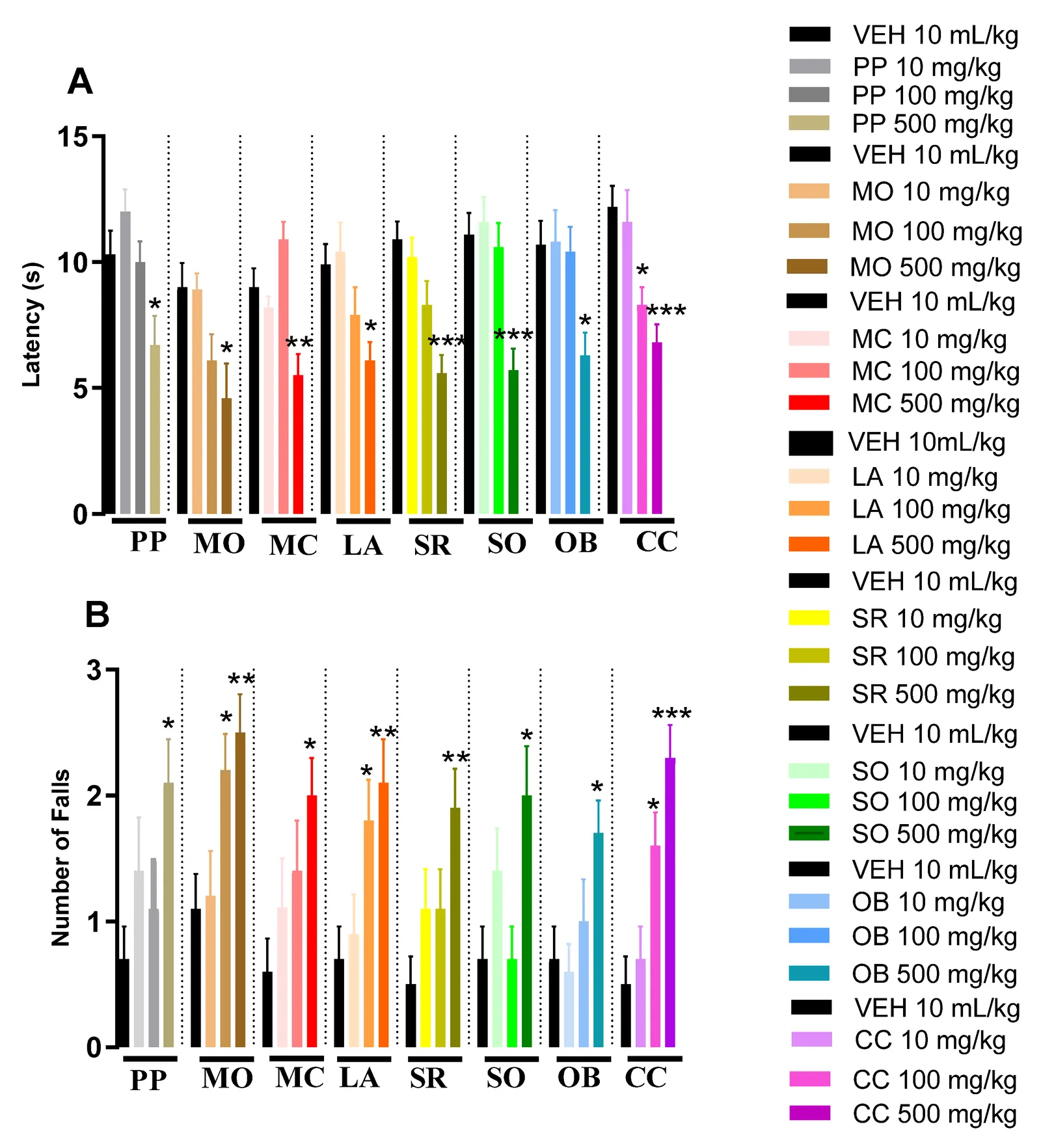
Effect of vehicle (VEH), EOs of *Pimenta pseudocaryophyllus* (PP), *Melissa officinalis* (MO), *Matricaria chamomilla* (MC), *Lippia alba* (LA), *Salvia rosmarinus* (SR), *Spiranthera odoratissima* (SO), *Ocimum basilicum* (OB), and *Cymbopogon citratus* (CC) in the rotarod test. The experiments were carried out in mice 60 min after oral treatment with VEH 10 mL/kg, PP, MO, MC, LA, SR, SO, OB, and CC at doses of 10, 100, and 500 mg/kg. The parameters evaluated were latency to the first fall (**A**) and the number of falls (**B**). Each column represents the mean ± S.E.M. (*n* = 10 Swiss mice per group; 5 males and 5 females). Statistical analysis was performed by one-way ANOVA followed by Dunnett’s post hoc test for multiple comparisons. * *p* < 0.05, ** *p* < 0.01, and *** *p* < 0.001 other treatment groups vs. vehicle treated group.

In the rotarod, statistically significant differences were observed among experimental groups (Detailed experimental data with diazepam are in Table S4). Figure 4 shows the effect of oral treatment with vehicle (10 mL/kg), EOs [*Pimenta pseudocaryophyllus*, PP; *Melissa officinalis*, MO; *Matricaria chamomilla*, MC; *Lippia alba*, LA; *Salvia rosmarinus*, SR; *Ocimum basilicum*, OB; *Spiranthera odoratissima*, SO; and *Cymbopogon citratus*, CC] on the first fall (A) and the number of falls (B) of mice exposed to the rotarod test. Unlike PP and OB which slightly decreased the first fall latency [PP: F_(6,56)_  =  5.20; OB − F_(6,56)_  =  4.38] and increased the number of falls of the animals [PP: F_(6,56)_  =  2.72; OB − F_(6,56)_  =  3.34] at the highest dose (*p* < 0.05), other EOs − MO, MC, LA, SR, SO, and CC—elicited more profound increase in the fall latency [MO: F_(6,56)_  =  4.31; MC: F_(6,56)_  =  9.99; LA: F_(6,56)_  =  4.03; SR: F_(6,56)_  =  8.75; SO: F_(6, 56)_  =  8.84; and CC: F_(6,56)_  =  8.15] and number of falls [MO: F_(6,56)_  =  5.17; MC: F_(6,56)_  =  3.00; LA: F_(6,56)_  =  4.69; SR: F_(6,56)_  =  3.81; SO: F_(6,56)_  =  3.87; and CC: F_(6,56)_  =  10.84] at intermediate and highest doses with *p* < 0.01 (100 and 500 mg/kg, respectively).

## 3. Discussion

Natural products are increasingly recognized for their anxiolytic potential [18]. However, rigorous evaluation of their safety profiles remains essential to ensure translational relevance and clinical applicability [19,20]. EOs represent a promising class of phytotherapeutics that modulate CNS function through multiple molecular targets [10,21]. Preclinical evidence indicates that constituents such as linalool, citral, limonene, eugenol, and apigenin exert anxiolytic-like effects by modulating GABAA receptors, serotonergic pathways, and attenuating glutamatergic transmission [22,23,24]. These mechanisms partially overlap with those of conventional anxiolytics, although phytochemicals typically exhibit broader and less selective pharmacological profiles.

The lipophilicity properties of EOs could facilitate blood–brain barrier permeability, dose-dependent anxiolytic-like effects, and motor impairment [25,26,27]. Motor incoordination is often associated with excessive potentiation of inhibitory neurotransmission or disruption of neuronal homeostasis. From a clinical perspective, the distinction of anxiolysis from nonspecific CNS depression and motor effects is critical. Current guidelines recommend selective serotonin reuptake inhibitors and serotonin-norepinephrine reuptake inhibitors as first-line treatments for anxiety disorders due to their favorable long-term safety profiles relative to benzodiazepines, which are associated with tolerance, dependence, and motor impairment [25,28,29,30]. In this context, identifying EOs with anxiolytic properties, or using them as adjunctive agents without compromising motor function, remains an important therapeutic objective. The EO compositions reported in the literature (Table S1) and identified in this study (Table S3) guided compound selection for in silico analysis. Compounds were prioritized based on their relative abundance in the EOs, recurrent occurrence across the investigated species, and reported pharmacological activity relevant to the study.

Based on these biological findings, our docking studies suggest that major EO constituents may interact with M1 or M2 receptors. Although these predictions do not necessarily establish functional activity, they provide a plausible framework for interpreting the observed behavioral effects and prioritizing candidate mechanisms for future validation [31,32]. Geranial was predicted to interact with muscarinic receptors M1 and M2, which are implicated in central cholinergic regulation of motor function. These interactions may contribute to altered motor performance, potentially by modulating acetylcholine signaling [33]. The interactions of neral with muscarinic receptors suggest a potential modulation of acetylcholine balance, which is also critical for motor activity [34]. The predicted interaction of β-caryophyllene with M2 may reflect a cholinergic influence on motor incoordination-like effects. These predictions highlight the complexity of EO pharmacology and the likelihood of multiple-system modulation in their effects.

The data from in vivo evaluations of EOs reveal a non-linear dose–response pattern. At 10 mg/kg, none of the EOs elicited significant anxiolytic-like effects, suggesting a subthreshold exposure. At 100 mg/kg, EOs from *Pimenta pseudocaryophyllus*, *Melissa officinalis*, *Matricaria chamomilla*, *Salvia rosmarinus*, *Spiranthera odoratissima*, and *Ocimum basilicum* exhibited anxiolytic-like effects without motor deficits, indicating a favorable separation of effects. In contrast, *Lippia alba* and *Cymbopogon citratus* exhibited concurrent anxiolytic-like and motor-impairing effects at 100 mg/kg. Hypothetically, this profile may be related to their citral-rich composition. Citral has been associated with enhanced inhibitory neurotransmission and dose-dependent motor impairment [35].

At 500 mg/kg, all EOs elicited marked motor impairment. This finding aligns with previous evidence associating enhanced inhibitory neurotransmission with impaired motor coordination [3,30]. The reduction in open-arm entries and time spent in the open arms, along with a significant change in total arm entries at 500 mg/kg, suggests reduced locomotion, motor incoordination, or other nonspecific effects. The doses used in this study are experimental in mice and should not be directly extrapolated to humans on a simple mg/kg basis. The primary purpose of testing the broader dose range was to characterize the separation, or lack thereof, between behavioral effects compatible with anxiolytic-like activity and impairment of motor coordination.

Analysis of representative constituents from the EOs suggests that the observed motor impairment may arise from simultaneous modulation of multiple targets associated with motor control. Several constituents appear to converge on cholinergic neurotransmission, a key regulator of movement. Geranial’s interaction with both M1 and M2 muscarinic receptors could disrupt the balance between postsynaptic cholinergic excitation and presynaptic autoreceptor-mediated feedback [14]. Such dual modulation may reduce the precision of cholinergic transmission within motor circuits. This could elicit psychomotor depression, ataxia, and reduce spontaneous activity [15]. Likewise, eucalyptol (1,8-cineole), the predominant constituent of the EO from *Salvia rosmarinus*, and chavibetol from *P. pseudo-caryophyllus*, predicted to be acetylcholinesterase inhibitors, could increase synaptic acetylcholine concentration. Although moderate enhancement of cholinergic signaling may be beneficial in some neurological conditions, excessive cholinergic activity can impair motor output [10,14,15].

Neral, present in *Melissa officinalis* and *Cymbopogon citratus* EOs, is predicted to interact with muscarinic receptors (M1-M3) as well as D2- and D4-like dopamine receptors [7]. Because motor control depends on the finely regulated interaction between dopaminergic and cholinergic neurotransmission within the basal ganglia, concurrent modulation of these systems could disrupt the balance required for normal movement, increasing the likelihood of motor incoordination. Similarly, linalool from *Lippia alba* and *Ocimum basilicum* EOs was predicted to influence dopamine transporter activity, D2 receptors, and M4 muscarinic receptors [35].

These predicted target interactions reinforce the concept that motor impairment associated with anxiolytic EOs is unlikely to result from the action of a single constituent or receptor. Instead, it probably reflects the integrated effects of multiple bioactive constituents acting on interconnected neurotransmitter systems that regulate movement. This pharmacological profile is consistent with the complex chemical composition of EOs and supports the hypothesis that therapeutic anxiolytic effects may coexist with dose-dependent motor adverse effects. This study has limitations. The in silico predictions provide mechanistic hypotheses but do not establish direct target binding or functional activity. Although EPM arm entries and rotarod performance were used to assess potential locomotor and motor confounding, an independent locomotor assay would more clearly distinguish anxiety-related changes in exploration from nonspecific alterations in spontaneous activity. Finally, although both sexes were included, the study was not prospectively powered to assess sex × treatment interactions, and the estrous cycle was not monitored. Altogether, this broad-range assessment is useful for initial screening and for detecting dose-limiting motor impairment.

## 4. Materials and Methods

### 4.1. Reagents, Standard Drugs, and Essential Oils

Standard chavibetol, linalool, β-caryophyllene, neral, geranial, and eucalyptol (Analytical-grade) were purchased from Sigma-Aldrich (St. Louis, MO, USA) and Cayman Chemical (Ann Arbor, MI, USA). Leaves of *Pimenta pseudocaryophyllus*, *Melissa officinalis*, *Matricaria chamomilla*, *Lippia alba*, *Salvia rosmarinus*, *Ocimum basilicum*, *Spiranthera odoratissima*, and *Cymbopogon citratus* were collected in the metropolitan region of Goiânia, Brazil, and dried in a forced-air oven at 33 °C. Voucher materials were deposited in the University Herbarium of the Conservation Unit (UFG 27159, 7380, 1039, 10050, 43206, 2350, 60010, and 98522, respectively). This material was ground and subjected to hydrodistillation for 2 h using a Clevenger-type apparatus. The essential oils (EOs) were dried over anhydrous sodium sulfate, stored in amber vials, and kept at −18 °C until use. Before oral administration, all EOs were protected from light and heat, stored under identical conditions, and prepared according to standardized laboratory procedures. The chemical composition of EOs was determined by gas chromatography-mass spectrometry (GC-MS) using a Shimadzu QP5050A system (Shimadzu Corporation, Kyoto, Japan) equipped with a CBP-5 fused silica capillary column (30 m × 0.25 mm, 0.25 μm). Helium was used as the carrier gas (1.0 mL/min), with electron ionization at 70 eV. The oven temperature was programmed from 60 to 246 °C at 3 °C/min, then heated to 270 °C at 10 °C/min, followed by a final hold of 5 min. Constituents were identified by comparing Kovats retention indices, calculated relative to C8-C32 n-alkanes, and mass spectra with published data. Major constituents identified in each EO were used to select representative compounds for subsequent in silico analyses.

### 4.2. Docking and MD Simulations

SwissTargetPrediction was used to identify potential targets for this set of molecules [36,37]. Molecular docking was performed with the main compounds of the EOs (Table S3), with the possibility of motor impairment. All structures of representative compounds present in all EOs (chavibetol, linalool, β-caryophyllene, neral, geranial, and eucalyptol) were drawn in KingDraw v. 3.0 software and then subjected to partial charge assignment using the AM1-BCC method in UCSF Chimera. Energy minimization was then performed using the AMBER ff14SB force field to obtain energetically minimized three-dimensional conformations. Next, the molecules were converted to MOL2* format for molecular docking studies. Muscarinic receptors M1 (PDB ID: 5CXV) and M2 (PDB ID: 3UON) were retrieved from the RCSB Protein Data Bank (https://www.rcsb.org/). During protein preparation, all co-crystallized ligands and other non-essential molecules (water and ions) were removed to expose the binding sites. Considering that muscarinic receptors are G protein-coupled receptors, docking simulations targeted the orthosteric site, corresponding to the acetylcholine-binding region. Subsequently, molecular interactions were performed using GOLD (Genetic Optimization for Ligand Docking) v. 2022.1.0 (CCDC) [38], implemented in the HERMES v.5.4.0 software package, to investigate the binding modes of the EO compounds. For each run, ligands were placed within the same region occupied by the previously co-crystallized ligands to ensure proper sampling of the binding site. The best poses were selected based on the ChemPLP scoring function and visual inspection of molecular interactions, allowing for a final ranking of the compounds against the predicted targets to gain insights into the main potential macromolecular target related to the activity observed in the experimental analyses. Two-dimensional interaction maps were generated using the Discovery Studio Visualizer.

MD simulations were carried out using Maestro^®^ v. 2023.1 (Schrödinger suite, academic license from D. E. Shaw Research, https://www.deshawresearch.com/resources.html, accessed on 14 March 2023), employing the OPLS_2005 force field. The system was prepared using the Protein Preparation Wizard and adjusted to physiological pH 7.4 using the PROPKA module to ensure appropriate protonation states of ionizable residues.

Each system was solvated in an orthorhombic box of explicit water using the TIP3P model, maintaining a minimum distance of 10 Å between the protein and the box boundaries. After energy minimization to remove steric clashes, the systems were equilibrated under NPT conditions (constant number of particles, pressure, and temperature) at 300 K and 1 bar. Subsequently, 100 ns molecular dynamics simulations were performed for each complex, with a trajectory interval of 100 ps, yielding approximately 1000 frames per simulation. Post-simulation analyses included the assessment of conformational stability through Root Mean Square Deviation (RMSD) plots for both protein and ligand along the trajectory, using the most representative structure of the ligand-target complex, determined by the cluster analysis module. In addition, interactions between the potential target and the best ligand (as determined by the docking protocol) were monitored using interaction diagrams and occupancy fraction plots, enabling identification of residues with the most persistent contacts. Furthermore, Root Mean Square Fluctuation (RMSF) of protein residues was calculated to assess local flexibility of the polypeptide chain in response to ligand binding, providing insights into dynamically flexible or stabilized regions throughout the molecular dynamics simulation. All procedures follow previously published studies by our research group [39,40].

### 4.3. In Vivo Evaluations

#### 4.3.1. Animals and Treatments

Male and female Swiss albino mice (6–8 weeks old, 30–35 g; *n* = 10 per group; 5 males and 5 females) were obtained from the Central Animal Facility of the Evangelical University of Goiás (UniEVANGÉLICA). Animals were housed under controlled conditions (22 ± 2 °C; 12 h light/dark cycle) with free access to food and water and acclimatized for seven days before testing. All experimental procedures were conducted in accordance with ethical guidelines for animal research and approved by the Institutional Animal Care and Use Committee (Protocol No. 004/23). The estrous cycle was not monitored in this study.

EOs were dissolved in dimethyl sulfoxide and administered orally (p.o.) at doses of 10, 100, and 500 mg/kg. These doses were selected as part of an initial screening strategy informed by preliminary experiments and prior pharmacological protocols involving orally administered extracts and EOs. This range was intended to include a low exploratory dose, an intermediate dose with expected biological activity, and a high dose to identify potential dose-limiting motor impairment. Therefore, the study was designed to evaluate anxiolytic-like activity and compare motor safety across EOs.

Control animals received the vehicle at 10 mL/kg (p.o.). The selection of dose and vehicle for solubilization procedures was based on preliminary experiments. Anxiolytic-like and motor activity were evaluated with widely used pharmacological assays. Behavioral assessments were performed by experienced observers blinded to treatment groups. At the end of the behavioral experiments, animals were euthanized in accordance with institutional animal welfare guidelines. All procedures were performed by trained personnel.

#### 4.3.2. Elevated Plus Maze

The Elevated Plus Maze (EPM), a validated model of anxiety-like behavior [19], consisted of two open arms (30 cm × 5 cm) and two closed arms (30 cm × 5 cm × 20 cm, with 20 cm high walls) extending from a central platform (5 cm × 5 cm). The apparatus was elevated 50 cm above the floor. Sixty minutes after oral administration of EOs or diazepam (DZP 1 mg/kg), mice were placed on the central platform facing a closed arm and allowed to explore for 5 min. Time spent in the open arms, the number of open arm entries, closed arm entries, and total entries were recorded as indices of anxiolytic-like behavior [41].

#### 4.3.3. Rotarod Test

Motor coordination was assessed using a rotarod apparatus (AVS Projetos, Ribeirão Preto, São Paulo, Brazil) consisting of a non-slip rod (3 cm diameter, 30 cm length) rotating at a constant speed of 10 rpm [42]. To ensure baseline motor competence, animals were trained on the apparatus for 2 min, 24 h before oral administration of EOs or diazepam (DZP 5 mg/kg). Following treatment, mice were re-exposed to the rotarod in a single 1-min test session. Motor performance was evaluated by recording the latency to first fall (s) and the total number of falls. The rotarod test is a well-established method for assessing motor coordination, balance, and motor function in rodents [43,44,45,46].

### 4.4. Statistical Analysis

Behavioral data were analyzed using parametric tests after verification of normality and homogeneity of variance. For each EO and behavioral endpoint, data were analyzed separately using one-way ANOVA followed by Dunnett’s post hoc test, with each dose compared against the corresponding vehicle-treated group. Before inferential analyses, the assumptions required for parametric testing were assessed. The distribution of the behavioral data was examined for normality, and homogeneity of variance was also verified. Statistical analyses were performed using GraphPad Prism (version 8.0). Outliers were identified using Grubbs’ test (α = 0.05). Data are presented as mean ± standard error of the mean (SEM), *n* = 10 Swiss mice per group (5 males and 5 females), and statistical significance was set at *p* < 0.05.

## 5. Conclusions

In silico docking and molecular dynamics analyses suggested that computationally prioritized constituents present in all EOs interacted with cholinergic receptors, especially M1 and M2 receptors. The EOs exhibited a non-linear dose–response pattern in vivo. The intermediate dose consistently elicited anxiolytic-like effects, with increased open-arm exploration. In contrast, the highest dose was frequently associated with reduced exploration (open arm entries and total arm entries) that coincided with marked impairment in rotarod performance.

## Data Availability

The data will be available upon request from the Editor-in-Chief.

## Supplementary Materials

The following supporting information can be downloaded at: https://www.mdpi.com/article/10.3390/ijms27198786/s1. References [47,48,49,50,51,52,53,54,55,56,57,58,59,60,61,62,63,64,65,66,67,68,69,70,71,72,73,74,75,76,77,78,79,80,81,82,83,84,85,86,87,88,89,90,91,92,93,94,95] are cited in the Supplementary Materials.

## Abbreviations

The following abbreviations are used in this manuscript:

Abbreviation	Definition
AChE	Acetylcholinesterase
AM1-BCC	Austin Model 1 with Bond Charge Corrections
ANOVA	Analysis of Variance
CB2	Cannabinoid Receptor Type 2
CC	*Cymbopogon citratus*
CCDC	Cambridge Crystallographic Data Centre
CNS	Central Nervous System
CNPq	Brazilian National Council for Scientific and Technological Development
D2	Dopamine Receptor D2
D4	Dopamine Receptor D4
DAT	Dopamine Transporter
EO	Essential Oil
EOs	Essential Oils
EPM	Elevated Plus Maze
FAPEG	Goiás State Research Foundation
GABA*_A_*	γ-Aminobutyric Acid Type A Receptor
GC–MS	Gas Chromatography–Mass Spectrometry
GOLD	Genetic Optimization for Ligand Docking
GPCR	G Protein-Coupled Receptor
ICL3	Third Intracellular Loop
LA	*Lippia alba*
M1–M4	Muscarinic Acetylcholine Receptors 1–4
MC	*Matricaria chamomilla*
MD	Molecular Dynamics
MO	*Melissa officinalis*
NPT	Constant Number of Particles, Pressure, and Temperature Ensemble
OB	*Ocimum basilicum*
PDB	Protein Data Bank
PP	*Pimenta pseudocaryophyllus*
p.o.	*Per os* (oral administration)
RMSD	Root Mean Square Deviation
RMSF	Root Mean Square Fluctuation
SEM	Standard Error of the Mean
SO	*Spiranthera odoratissima*
SR	*Salvia rosmarinus*
TM6	Transmembrane Helix 6
VEH	Vehicle
