# Supplementary material for: Anxiolytic Potential of Selected Essential Oils and Their Impact on Motor Function
Source: Int J Mol Sci. 2026 Sep 30;27(19):8786. doi: 10.3390/ijms27198786 (PMC13664909; doi:10.3390/ijms27198786)
Supplement: Supplementary file 1 [file ijms-27-08786-s001.zip › ijms-4596484-supplementary.pdf]

Table S1. Selected sources of essential oils and active principles

Species / References	*Composition
Pimenta pseudocaryophyllus [47–63]	α-copaene (0.4% – RI = 1373), methyl eugenol (1.6% – RI = 1398), (E)-caryophyllene (1.7% – RI = 1416), (Z)-methyl isoeugenol (2.0% RI = 1451), δ-cadinene (0.4% – RI = 1521) and **(E)-methyl isoeugenol (93.9% – RI = 1494)**<br>Geranial (34.26%), neral (27.85%) and eugenol (71.9%)<br>Eucalyptol (14.2%) and limonene (9.3%)<br>Chavibetol (70.9%)<br>Asarone (as β-asarone) (~21.8%)
Melissa officinalis [57,64–67]	Oxygenated monoterpenes represented the most abundant compound class (84.5%) with citronellal (22.2%), neral (17.9%), and geranial (23.8%) as the major essential-oil constituents<br>Germacrene D (32.08%)<br>Caryophyllene oxide (27.06%)<br>Geranial (44.00%)
Matricaria chamomilla [54,55,68–70]	α-pinene (0.36%, RI = 930), Camphene (<0.1%, RI = 956), Butyl butyrate (0.14%, RI = 998), para-Cymene (0.25%, RI = 1023), n-Octanol (0.12%, RI = 1049), Linalool (0.18%, RI = 1086), Borneol (0.29%, RI = 1120), para-Cymene-8-ol (0.12%, RI = 1135) 3-Decanol (0.20%, RI = 1142), Ethyl octanoate (0.17%, RI = 1154), Hexyl 3-methyl butanoate (0.40%, RI = 1173), Trans-anethol (0.36%, RI = 1205), Azulene (0.14%, RI = 1226), β-Elemene (0.12%, RI = 1260), β-Caryophyllene (1.10%, RI = 1277), γ-Elemene (0.70%, RI = 1283), Trans-β-farnesene (5.36%, RI = 1342), Germacrene D (0.31%, RI = 1349), α-muurolene (0.65%, RI = 1358), γ-Cadinene (0.22%, RI = 1365), Spathulenol (8.83%, RI = 1422), Caryophyllene oxide (0.37%, RI = 1565), Viridiflorol (1.59%, RI = 1588), γ-Eudesmol (2.10%, RI = 1623), α-Bisabolol oxide B (9.35%, RI = 1650), α -Bisabolol (10.94%, RI = 1696), α-Bisabolone oxide A (9.82%, RI = 1743), α -Bisabolol oxide A (30.42%, RI = 1752), Chamazulene (2.40%, RI = 1773), cis-en-yn-Dicycloether (6.94%, RI = 1890), trans-en-yn-dicycloether (1.28%, RI = 1912)<br>α-Bisabolol (27.36–38.6%)<br>α-Bisabolol oxide A and B (7.31–51.31%)<br>α-Bisabolone oxide A (45.64–65.41%)<br>Chamazulene (31.2%)<br>β-Farnesene (25.05–30.15%)<br>Eucalyptol (8.19%) and D-limonene (15.25%)
[71]	α-bisabolol oxide A (28%), α-bisabolol oxide B (17.1%), (Z)-β-Farnesene (15.9%) and α-bisabolol (6.8%) as the main components
Lippia alba [71–78]	Geranial (20.7–50.9%); Neral (16.4–33.3%)<br>Linalool (46.5–90.7%)<br>Carvone (often L-carvone) (~37%)<br>β-Myrcene (3.96–19.49%)<br>carvone(54.57%,RI = 1244), limonene (23.13%, RI = 1005), alpha-murolene (4.84%, RI = 1490), sabinene (2.98%, 956), bulnesol (2.14%, RI = 1564)
[79]	Linalool (81.64%, RI = 1099), Eucalyptol (3.82%, RI = 1030), α-Citral (2.30%, RI =1266), β-Cubebene (1.87%, RI = 1480), β-Caryophyllene (1.60%, RI = 1418), Carveol (1.39%, RI = 1207)
Salvia rosmarinus [80–84]	1,8-Cineole (eucalyptol) (monoterpene oxide) (43.77%)<br>α-Pinene (13.5%–37.7%)<br>**Camphor** (oxygenated monoterpene ketone) (21.9%)<br>1,8 cineole (37.6%, RI = 1034), α-pinene (19.9%, RI = 935), and linalool (15.8%, RI = 1090),Camphene (2.6%, RI = 952),Myrcene (2.54%, RI = 983), Limonene (4.12, RI = 102), Camphor (2.8%, RI = 1125), Borneol (3.6%, RI = 1170), α-terpineol (2.4%, RI = 1183), Bornyl acetate (1.9%, RI = 1276), β-caryophyllene (1.65%, RI = 1422), β-farnesene (1.18%, RI = 1454)
[85]	eucalyptol (26.02%, RI = 1033), α-pinene (19.89%, RI = 934), camphor (16.71%, RI = 1148), camphene

	(8.67%, RI = 949), β-myrcene (3.97%, RI = 988), β-caryophyllene (3.11%), borneol (2.50%), and limonene (2.16%)
Spiranthera odoratissima [86]	β-caryophyllene (20.64%), gamma-muurolene (17.7%), bicyclogermacrene (14.73%), delta-cadinene (13.40%), gamma-cadinene (4.59%), and cubenol (3.12%)
Ocimum basilicum [87]	methyl chavicol (42.8%), geranial (13.0%), neral (12.2%), and β-caryophyllene (7.2%)
[88]	linalool (31 %), camphor, β-elemene, α-bergamotene, and bornyl-acetate, estragole (15.57%), eugenol (2.64%), and 1,8-cineole (3.29%)
[89]	Linalool L (35.945%), 1,8-Cineole (11.228%), Alpha-cadinol (10.395%), Farnesyl acetate (10.178%), E,E-alpha-farnesene (4.851%), Trans-beta-ocimene (3.724%), Gamma cadinene (3.101%), (-)-Camphor (2.331%), Borneol (1.96%), Alpha-copaene (1.75%), Fenchyl acetate (1.316%), L-alpha-bornyl acetate (1.225%), Cis-beta-terpineol (1.19%), Alpha-guaiene (1.162%)
Cymbopogon citratus [90]	geranial (40.8%), neral (36.3%), and beta-myrcene (13.2%)
[91]	citral (71.29%), a mixture of the stereoisomers geranial and neral, or β-myrcene (16.5%), geraniol (1.28%), 6-methyl-5-hepten-2-ona (0.64%), and undecan-2-one (0.29%)
[92]	myrcene, Z-geranial, E-geranial account for 10.7%, 32.1%, and 32.9%, respectively
[93]	geranial (52.1%), neral (35.2%), myrcene (4.1%)
[94]	geranial (41.84%), neral (31.49%), Geranyl acetate (9.04%), Geraniol (6.0%)

Table S2. Ranking of compounds based on Chem(PLP) scores for M1 and M2 receptors

Receptor	Compound	ChemPLP
M1	Chavebitol	61.70
	Linalool	58.14
	β-Caryophyllene	55.50
	Neral	42.93
	Geranial	39.66
	Eucalyptol	8.59
M2	Linalool	61.14
	Chavebitol	60.95

β-Caryophyllene	53.32
Geranial	37.48
Neral	36.01
Eucalyptol	18.09

Tables S3. Chemical composition of essential oils as determined by GC–MS.

		Area (%)							
KI	Compounds	PP	MO	MC	LA	SR	OB	SO	CC
932	*α*-Pinene	-	-	6.77	0.70	13.92	5.44	-	0.64
974	*β*-Pinene	-	-	6.55	4.55	0.31	-	-	0.38
988	Myrcene	-	-	0.37	8.02	2.05	12.24	-	13.60
1002	*α*-Phellandrene	-	-	-	-	1.99	-	-	1.99
1025	*β*-Phellandrene	-	-	-	-	5.94	-	-	-
1029	Limonene	-	-	10.13	40.10	-	-	-	2.05
1031	Eucalyptol (1,8-cineole)	*3.66*	2.23	5.95	6.56	23.43	6.04	5.61	5.99
1037	Z-*β*-Ocimene	-	-	-	-	0.58	-	-	0.66
1048	E-*β*-Ocimene	-	-	-	-	0.28	-	-	-
1097	Linalool	3.88	3.92	3.47	11.48	3.64	33.06	3.26	3.33
1143	Camphor	-	-	-	-	10.01	-	-	-
1177	Terpinen-4-ol	-	-	-	-	0.43	0.43	-	-
1184	Cryptone	-	-	-	-	4.78	4.78	-	-
1189	α-Terpineol	-	-	-	-	0.82	0.82	-	-
1235	Neral	*6.18*	5.76	0.45	8.01	0.95	0.66	0.85	14.23
1240	Cuminaldehyde	-	-	-	-	0.44	2.36	-	
1251	Geranial	8.45	9.13	7.99	10.82	7.12	8.01	7.98	21.86
1290	Thymol	-	-	-	-	0.38	-	-	-
1330	3-oxo-*p*-Menth-1-em-7-al	-	-	-	-	0.39	-	-	-
1338	*δ*-Elemene	-	-	-	-	-	-	2.16	-
1351	*α*-Cubebene	-	-	-	-	-	-	0.30	-
1366	Chavibetol	11.22	6.27	11.99	2.04	3.76	2.43	11.77	3.92
1374	*α*-Copanene	-	-	-	-	-	0.25	2.64	-
1391	*β*-Elemene	-	-	-	-	-	-	4.19	-
1403	Methyl eugenol	20.34	10.34	-	-	-	10.02	-	-

1418	*β*-Caryophyllene	4.70	17.02	4.92	6.08	4.52	4.94	4.55	5.02
1432	*trans*-*α*-Bergamotene	-	-	-	-	-	-	-	-
1441	Aromadendrene	-	-	-	-	-	-	0.44	0.29
1450	*cis*-Muurola-3,5-diene	-	-	-	-	-	-	0.18	4.14
1454	*α*-Humulene	-	-	0.52	0.52	4.61	1.54	2.25	0.54
1452	Ethyl vanillin	-	-	0.45	0.45	-	-	-	4.52
1456	β-Farnesene	-	-	2.44	-	-	-	-	-
1458	*allo*-Aromadendrenen	-	-	-	-	-	-	4.00	4.60
1476	trans-Cadina-1(6),4-diene	-	-	-	-	-	-	0.66	-
1479	*γ*-Muurolene	-	-	-	-	-	0.88	1.76	-
1481	Germacrene D	-	8.05	-	-	-	-	4.00	-
1489	*β*-Selinene	-	-	-	-	0.88	-	-	1.83
1492	E-Methyl eugenol	18.46	28.11	-	-	-	4.56	-	-
1493	*δ*-Selinene	-	-	-	-	1.8	1.2	-	2.66
1495	trans-Muurola-4(14),5-diene	-	-	-	-	-	-	0.54	-
1500	Bicyclogermacrene	-	-	-	-	-	-	11.29	0.44
1500	*α*-Muurolene	-	-	-	-	-	-	4.14	0.18
1509	Germacrene A	-	-	-	-	-	-	0.54	2.25
1513	*γ*-Cadinene	-	-	-	-	-	-	4.52	-
1523	δ-Cadinene	-	-	-	-	-	-	10.60	0.40
1534	*trans*-Cadina-1,4-diene	-	-	-	-	-	-	0.61	1.72
1538	*α*-Cadinene	-	-	-	-	-	-	1.12	1.27
1557	Elemicin	25.80		-	-	-	-	-	2.48
1583	Caryophyllene oxide	-	2.02	-	-	6.56	-	-	-
1590	*β*-Copaen-4-*α*-ol	-	-	-	-	-	-	0.74	-
1590	Globulol	-	-	-	-	-	-	0.81	-
1592	Viridiflorol	-	-	-	-	-	-	0.77	-
1600	Guaiol	-	-	-	-	-	-	0.42	-
1608	Humulene epoxide II	-	-	-	-	0.9	-	-	-
1628	1-epi-Cubenol	-	-	-	-	-	-	0.40	-
1645	*α*-Muurolol	-	0.38	-	-	-	-	6.72	-
1646	Cubenol	-	-	-	-	-	-	1.27	-
1658	Valerianol	-	-	-	-	-	0.9	8.48	-
1680	Asarone	0.34	-	-	-	-	-	-	
1683	α-Bisabolol	-	-	15.45	-	-	-	-	-
1667	14-hydroxy-Z-Caryophyllene	-	-	-	-	0.34	0.38	-	1.44

1670	Bisabolol oxide B	-	-	8.23	-	-	-	-	-
1713	Bisabolol oxide A	-	-	7.54	-	-	-	-	-
1735	Chamazulene	-	-	6.23	-	-	-	-	-

KI, Kovatz Index; *Pimenta pseudocaryophyllus* (PP), *Melissa officinalis* (MO), *Matricaria chamomilla* (MC), *Lipia alba* (LA), *Salvia rosmarinus* (SR), *Spiranthera odoratissima* (SO), *Ocimum basilicum* (OB), and *Cymbopogon citratus* (CC).

Table S3. Evaluation of EOs and diazepam in the elevated plus maze

Treatment groups	Dose	Number of open arm entries	Time spent in the open arms	Number of closed arm entries	Total entries
VEH	10 mL/kg	40 ± 2.2	82 ± 4.2	22 ± 2.6	62 ± 3.4
PP	10 mg/kg	45 ± 3.3	94 ± 3.3	14 ± 1.2	59 ± 3.5
PP	100 mg/kg	60 ± 4.3	111 ± 3.0	16 ± 3.0	76 ± 5.2
PP	500 mg/kg	28 ± 3.0	87 ± 3.4	17 ± 4.0	45 ± 5.0
DZP	1 mg/kg	66 ± 1.8	121 ± 2.4	12 ± 3.6	78 ± 4.4
		Number of open arm entries	Time spent in the open arms	Number of closed arm entries	Total entries
VEH	10 mL/kg	50 ± 4.2	85 ± 2.2	20 ± 1.6	70 ± 5.6
MO	10 mg/kg	55 ± 2.1	90 ± 2.1	12 ± 2.6	67 ± 3.3
MO	100 mg/kg	68 ± 3.3	100 ± 3.2	18 ± 3.6	86 ± 5.9
MO	500 mg/kg	34 ± 3.1	84 ± 3.1	16 ± 2.0	50 ± 3.7
DZP	1 mg/kg	70 ± 2.1	118 ± 1.4	14 ± 2.4	84 ± 4.6
		Number of open arm entries	Time spent in the open arms	Number of closed arm entries	Total entries
VEH	10 mL/kg	46 ± 2.7	85 ± 5.0	13 ± 2.6	59 ± 3.7
MC	10 mg/kg	45 ± 4.0	88 ± 3.3	11 ± 3.6	56 ± 5.4
MC	100 mg/kg	66 ± 5.0	110 ± 3.1	17 ± 1.4	83 ± 5.2
MC	500 mg/kg	30 ± 4.0	99 ± 4.0	20 ± 3.8	50 ± 5.5
DZP	1 mg/kg	68 ± 2.1	114 ± 2.0	18 ± 1.0	86 ± 2.3
		Number of open arm entries	Time spent in the open arms	Number of closed arm entries	Total entries
VEH	10 mL/kg	40 ± 2.2	78 ± 3.0	25 ± 5.0	65 ± 5.5
LA	10 mg/kg	46 ± 3.0	83 ± 3.4	11 ± 2.0	57 ± 3.6
LA	100 mg/kg	55 ± 4.0	95 ± 3.2	13 ± 1.1	68 ± 4.1
LA	500 mg/kg	28 ± 2.2	86 ± 2.1	8 ± 1.6	36 ± 2.7
DZP	1 mg/kg	70 ± 1.8	100 ± 3.0	10 ± 5.0	80 ± 5.3

		**Number of open arm entries**	**Time spent in the open arms**	**Number of closed arm entries**	**Total entries**
VEH	10 mL/kg	46 ± 2.7	98 ± 3.4	21 ± 4.0	67 ± 4.8
SR	10 mg/kg	47 ± 3.3	100 ± 3.1	15 ± 3.4	62 ± 4.7
SR	100 mg/kg	60 ± 3.3	112 ± 3.3	17 ± 1.8	77 ± 3.8
SR	500 mg/kg	35 ± 2.4	84 ± 3.5	10 ± 2.5	45 ± 3.5
DZP	1 mg/kg	74 ± 5.1	120 ± 1.8	8 ± 2.8	82 ± 5.8
		**Number of open arm entries**	**Time spent in the open arms**	**Number of closed arm entries**	**Total entries**
VEH	10 mL/kg	47 ± 4.4	86 ± 4.1	17 ± 3.0	64 ± 5.3
SO	10 mg/kg	52 ± 3.0	87 ± 3.4	10 ± 2.4	62 ± 3.8
SO	100 mg/kg	61 ± 4.2	100 ± 2.2	15 ± 1.3	76 ± 4.4
SO	500 mg/kg	46 ± 2.1	70 ± 4.0	8 ± 3.3	54 ± 3.9
DZP	1 mg/kg	68 ± 3.4	116 ± 2.8	11 ± 3.5	79 ± 4.9
		**Number of open arm entries**	**Time spent in the open arms**	**Number of closed arm entries**	**Total entries**
VEH	10 mL/kg	41 ± 3.4	80 ± 2.9	8 ± 1.5	49 ± 3.7
OB	10 mg/kg	47 ± 3.1	90 ± 2.1	11 ± 2.8	58 ± 4.2
OB	100 mg/kg	64 ± 3.3	99 ± 3.4	14 ± 2.3	78 ± 4.0
OB	500 mg/kg	52 ± 2.4	84 ± 3.1	8 ± 1.0	60 ± 2.6
DZP	1 mg/kg	66 ± 1.4	110 ± 1.8	15 ± 2.7	81 ± 3.0
		**Number of open arm entries**	**Time spent in the open arms**	**Number of closed arm entries**	**Total entries**
VEH	10 mL/kg	50 ± 4.3	86 ± 3.7	24 ± 4.7	74 ± 6.4
CC	10 mg/kg	47 ± 3.0	86 ± 3.3	12 ± 3.4	59 ± 4.5
CC	100 mg/kg	66 ± 4.2	109 ± 3.4	20 ± 4.0	86 ± 5.8
CC	500 mg/kg	30 ± 2.1	66 ± 3.1	10 ± 2.1	40 ± 3.0
DZP	1 mg/kg	75 ± 2.4	112 ± 1.4	12 ± 3.0	87 ± 3.8

Table S4. Evaluation of EOs and diazepam in the rotarod test

**Treatment groups**	**Dose**	**Latency to first fall**	**Number of falls**
VEH	10 mL/kg	10 ± 0.9	0.7 ± 0.2
PP	10 mg/kg	12 ± 0.8	1.4 ± 0.4
PP	100 mg/kg	10 ± 0.8	1.1 ± 0.4

PP	500 mg/kg	7 ± 1.2	2.1 ± 0.3
DZP	5 mg/kg	3 ± 0.7	4.2 ± 0.2
		**Latency to first fall**	**Number of falls**
VEH	10 mL/kg	9 ± 1.0	1.1 ± 0.3
MO	10 mg/kg	8 ± 0.7	1.2 ± 0.4
MO	100 mg/kg	6 ± 1.0	2.2 ± 0.3
MO	500 mg/kg	5 ± 1.4	2.5 ± 0.3
DZP	1 mg/kg	3 ± 0.8	4.4 ± 0.4
		**Latency to first fall**	**Number of falls**
VEH	10 mL/kg	9 ± 0.8	0.6 ± 0.3
MC	10 mg/kg	8 ± 0.4	1.1 ± 0.4
MC	100 mg/kg	11 ± 0.7	1.4 ± 0.4
MC	500 mg/kg	5 ± 0.9	2.0 ± 0.3
DZP	5 mg/kg	2 ± 0.6	4.0 ± 0.5
		**Latency to first fall**	**Number of falls**
VEH	10 mL/kg	10 ± 0.8	0.7 ± 0.3
LA	10 mg/kg	10 ± 1.2	0.9 ± 0.3
LA	100 mg/kg	8 ± 1.1	1.8 ± 0.3
LA	500 mg/kg	6 ± 0.7	2.1 ± 0.3
DZP	5 mg/kg	3 ± 0.2	4.0 ± 0.5
		**Latency to first fall**	**Number of falls**
VEH	10 mL/kg	11 ± 0.7	0.5 ± 0.2
SR	10 mg/kg	10 ± 0.8	1.1 ± 0.3
SR	100 mg/kg	8 ± 0.9	1.1 ± 0.3
SR	500 mg/kg	6 ± 0.7	1.9 ± 0.3
DZP	5 mg/kg	2 ± 0.4	3.8 ± 0.2
		**Latency to first fall**	**Number of falls**
VEH	10 mL/kg	11 ± 0.9	0.7 ± 0.3
SO	10 mg/kg	12 ± 1.0	1.4 ± 0.3
SO	100 mg/kg	11 ± 1.0	0.7 ± 0.3
SO	500 mg/kg	6 ± 0.9	2.0 ± 0.4
DZP	5 mg/kg	3 ± 0.6	3.3 ± 0.6
		**Latency to first fall**	**Number of falls**
VEH	10 mL/kg	11 ± 0.9	0.7 ± 0.3
OB	10 mg/kg	11 ± 1.3	0.6 ± 0.2
OB	100 mg/kg	10 ± 1.0	1.0 ± 0.3
OB	500 mg/kg	6 ± 0.9	1.7 ± 0.3
DZP	5 mg/kg	4 ± 0.2	3.9 ± 0.4

		Latency to first fall	Number of falls
VEH	10 mL/kg	12 ± 0.8	0.5 ± 0.2
CC	10 mg/kg	12 ± 1.2	0.7 ± 0.3
CC	100 mg/kg	8 ± 0.7	1.6 ± 0.3
CC	500 mg/kg	7 ± 0.7	2.3 ± 0.3
DZP	5 mg/kg	2 ± 0.4	3.0 ± 0.2

References

47. Patel, D.N.; Ho, H.K.; Tan, L.L.; Tan, M.B.; Zhang, Q.; Low, M.; Chan, C.; Koh, H. Hepatotoxic Potential of Asarones : In Vitro Evaluation of Hepatotoxicity and Quantitative Determination in Herbal Products. **2015**, *6*, 1–13, doi:10.3389/fphar.2015.00025.
48. Fajemiroye, J.O.; Galdino, P.M.; Alves, S.F.; Paula, J.A.M. de; Paula, J.R. de; Ghedini, P.C.; Costa, E.A. Involvement of 5-HT1A in the Anxiolytic-like Effect of Dichloromethane Fraction of Pimenta Pseudocaryophyllus. *J. Ethnopharmacol.* **2012**, *141*, 872–877, doi:https://doi.org/10.1016/j.jep.2012.03.043.
49. Marques, F.A.; Wendler, E.P.; Baroni, A.C.M.; Oliveira, P.R. de; Sasaki, B.S.; Guerrero Jr, P.G. Leaf Essential Oil Compositon of Pimenta Leaf Essential Oil Compositon of Pimenta Pseudocaryophyllus (Gomes) L . R . Landrum Native From Brazil. *J. Essent. Oil Res.* **2011**, *22*, 150–152, doi:10.1080/10412905.2010.9700289.
50. Niculau, E. dos S.; Ribeiro, L. do P.; Ansante, T.F.; Fernandes, J.B.; Forim, M.R.; Vieira, P.C.; Vendramim, J.D.; Silva, M.F. das G.F. da Isolation of Chavibetol and Methyleugenol from Essential Oil of Pimenta Pseudocaryophyllus by High Performance Liquid Chromatography. *Molecules* **2018**, *23*, 2909, doi:10.3390/molecules23112909.
51. Ribeiro, L. do P.; Faria, · Rodrigo Donizeti Nicolau · Edenilson dos Santos; Padoan, · Gabriel Luiz; Gonçalves; Ansante, T.F.; da Silva, M.F. das G.F.; Vendramim, J.D. Essential Oil from Pimenta Pseudocaryophyllus (Myrtaceae) Leaves and Its Major Compounds Against the Mexican Bean Weevil. *Gesunde Pflanze* **2024**, *76*, 65–73, doi:10.1007/s10343-023-00950-0.
52. Barata, L.E.S.; Santos, B.C.B.; Marques, F.A.; Baroni, A.C.M.; Oliveira, P.R. De; Einloft, P.; Ribeiro, J.C.L.; Guerrero Jr, P.G. Seasonal Variation of the Volatile Constituents from Leaves of Pimenta Pseudocaryophyllus (Gomes). *J. Essent. Oil Res.* **2011**, *23*, 54–57, doi:10.1080/10412905.2011.9700469.
53. Kemprai, P.; Bora, P.K.; Saikia, S.P.; Haldar, S. Chavibetol: Major and Potent Phytotoxin in Betel (Piper Betle L.) Leaf Essential Oil. *Pest Manag. Sci.* **2023**, *79*, 4451–4462, doi:https://doi.org/10.1002/ps.7645.
54. Kim, Y.W.; Kim, M.J.; Chung, B.Y.; Bang, D.Y.; Lim, S.K.; Choi, S.M.; Lim, D.S.; Cho, M.C.; Yoon, K.; Kim, H.S.; et al. Safety Evaluation and Risk Assessment of D-Limonene. *J. Toxicol. Environ. Health. B. Crit. Rev.* **2013**, *16*, 17–38, doi:10.1080/10937404.2013.769418.
55. Kumar, K.J.; Sonnathi, S.; Anitha, C.; Santhoshkumar, M. Eucalyptus Oil Poisoning. *Toxicol. Int.* **2015**, *22*, 170–171, doi:10.4103/0971-6580.172259.
56. Quentin, T.; Franke, H.; Lachenmeier, D.W. Risk Assessment of Isoeugenol in Food Based on Benchmark Dose-Response Modeling. *Toxics* **2023**, *11*, doi:10.3390/toxics11120991.
57. Custódio, D.L.; Burgo, R.P.; Moriel, B.; Melo, A. De; Bianchini, E.; Faria, T.D.J. Antimicrobial Activity of Essential Oils from Pimenta Pseudocaryophyllus and Tynanthus Micranthus. *BRAZILIAN Arch. Biol. Technol.* **2010**, *53*, 1363–1369.
58. Maranho, L.T.; Rosas, E.C.; Costa, T.A.; Luiz, J.; Ribas, C.; Filho, F.B.; Gabardo, M.C.L. Evaluation of the Chemical Composition and Oral Antimicrobial Activity of the Essential Oil from the Leaves of Pimenta (Gomes) Landrum. *Pharmacognosy Res.* **2020**, *12*, 65–70, doi:10.4103/pr.pr.

59. Santos, B.C.B. dos; Silvaa, J.C.T. da; Guerrero Jr, P.G.; Leitão, G.G.; Barata, L.E.S. Isolation of Chavibetol from Essential Oil of Pimenta Pseudocaryophyllus Leaf by High-Speed Counter-Current Chromatography. *J. Chromatogr. A* **2009**, *1216*, 4303–4306, doi:10.1016/j.chroma.2009.01.111.
60. Paula, J.A.M.; Ferri, P.H.; Bara, M.T.F.; Tresvenzol, L.M.F.; Sá, F.A.S.; Paula, J.R. Infraspecific Chemical Variability in the Essential Oils of Pimenta Pseudocaryophyllus (Gomes) L.R. Landrum (Myrtaceae). *Biochem. Syst. Ecol.* **2011**, *39*, 643–650, doi:10.1016/j.bse.2011.05.013.
61. Paula, J.A.M. de; Silva, M. do R.R.; Costa, M.P.; Diniz, D.G.A.; .Sá, F.A.. .; Alves, S.F.; Costa, E.A.; Lino, R.C.; Paula, J.R. De Phytochemical Analysis and Antimicrobial, Antinociceptive, and Anti-Inflammatory Activities of Two Chemotypes of Pimenta Pseudocaryophyllus (Myrtaceae). *Evidence-based Complement. Altern. Med.* **2012**, *2012*, 420715, doi:10.1155/2012/420715.
62. Paula, J.A.M. De; Paula, J.R. De; Bara, M.T.F.; Ferri, P.H.; Santos, S.C.; Silva, H.S.; Paula, J.R. De; Bara, M.T.F.; Ferri, P.H.; Santos, S.C. Chemical Differences in the Essential Oil of Pimenta Pseudocaryophyllus (Gomes) L . R . Landrum Leaves from Brazil. *J. Essent. Oil Res.* **2011**, *22*, 555–557, doi:10.1080/10412905.2010.9700398.
63. YOKOMIZO, N.K.S.; NAKAOKA-SAKITA, M. Atividade Antimicrobiana e Rendimento Do Óleo Essencial de Pimenta Pseudocaryophyllus Var . Pseudocaryophyllus (Gomes) Landrum , Myrtaceae. *Rev. Bras. Plantas Med.* **2014**, *16*, 513–520.
64. Delgado, C.; Mendez-Callejas, G.; Celis, C. Caryophyllene Oxide, the Active Compound Isolated from Leaves of Hymenaea Courbaril L. (Fabaceae) with Antiproliferative and Apoptotic Effects on PC-3 Androgen-Independent Prostate Cancer Cell Line. *Molecules* **2021**, *26*, doi:10.3390/molecules26206142.
65. Montalvão, M.M.; Felix, F.B.; Propheta Dos Santos, E.W.; Santos, J.F.; de Lucca Júnior, W.; Farias, A.S.; de Souza Ribeiro, A.; Cavaleiro, C.; Machado, S.M.F.; Scher, R.; et al. Cytotoxic Activity of Essential Oil from Leaves of Myrcia Splendens against A549 Lung Cancer Cells. *BMC Complement. Med. Ther.* **2023**, *23*, 139, doi:10.1186/s12906-023-03969-y.
66. Souihi, M.; Amri, I.; Souissi, A.; Hosni, K.; Brahim, N. Ben; Annabi, M. Essential Oil and Fatty Acid Composition of Melissa Officinalis L. *Prog. Nutr.* **2020**, *22*, 253–258, doi:10.23751/pn.v22i1.7758.
67. Stojanović, N.M.; Mladenović, M.Z.; Maslovarić, A.; Stojiljković, N.I.; Randjelović, P.J.; Radulović, N.S. Lemon Balm (Melissa Officinalis L.) Essential Oil and Citronellal Modulate Anxiety-Related Symptoms – In Vitro and in Vivo Studies. *J. Ethnopharmacol.* **2022**, *284*, 114788, doi:https://doi.org/10.1016/j.jep.2021.114788.
68. Alvarado-García, P.A.A.; Soto-Vásquez, M.R.; Rodrigo-Villanueva, E.M.; Gavidia-Valencia, J.G.; Rodríguez, N.M.G.; Rengifo-Penadillos, R.A.; Campos-Florián, J.V.; Guzmán, Y.E.R. de Chamomile (Matricaria Chamomilla L.) Essential Oil and Its Potential Against Stress, Anxiety, and Sleep Quality. *Pharmacogn. J.* **2024**, *16*.
69. El Mihyaoui, A.; Esteves da Silva, J.C.G.; Charfi, S.; Candela Castillo, M.E.; Lamarti, A.; Arnao, M.B. Chamomile (Matricaria Chamomilla L.): A Review of Ethnomedicinal Use, Phytochemistry and Pharmacological Uses. *Life (Basel, Switzerland)* **2022**, *12*, doi:10.3390/life12040479.
70. Rizvi, S.A.H.; Ling, S.; Tian, F.; Xie, F.; Zeng, X. Toxicity and Enzyme Inhibition Activities of the Essential Oil and Dominant Constituents Derived from Artemisia Absinthium L. against Adult Asian Citrus Psyllid Diaphorina Citri Kuwayama (Hemiptera: Psyllidae). *Ind. Crops Prod.* **2018**, *121*, 468–475, doi:https://doi.org/10.1016/j.indcrop.2018.05.031.
71. Can, Ö.D.; Demir Özkay, Ü.; Kıyan, H.T.; Demirci, B. Psychopharmacological Profile of Chamomile (Matricaria Recutita L.) Essential Oil in Mice. *Phytomedicine* **2012**, *19*, 306–310, doi:https://doi.org/10.1016/j.phymed.2011.10.001.
72. Oliveira, D.R.; Leitão, G.G.; Santos, S.S.; Bizzo, H.R.; Lopes, D.; Alviano, C.S.; Alviano, D.S.; Leitão, S.G. Ethnopharmacological Study of Two Lippia Species from Oriximiná, Brazil. *J. Ethnopharmacol.* **2006**, *108*, 103–108, doi:https://doi.org/10.1016/j.jep.2006.04.018.

73. Siani, A.C.; Tappin, M.R.R.; Ramos, M.F.S.; Mazzei, J.L.; Ramos, M.C.K. V; De Aquino Neto, F.R.; Frighetto, N. Linalool from Lippia Alba: Study of the Reproducibility of the Essential Oil Profile and the Enantiomeric Purity. *J. Agric. Food Chem.* **2002**, *50*, 3518–3521, doi:10.1021/jf011592n.
74. Belsito, D.; Bickers, D.; Bruze, M.; Calow, P.; Greim, H.; Hanifin, J.M.; Rogers, A.E.; Saurat, J.H.; Sipes, I.G.; Tagami, H. A Toxicologic and Dermatologic Assessment of Cyclic and Non-Cyclic Terpene Alcohols When Used as Fragrance Ingredients. *Food Chem. Toxicol.* **2008**, *46*, S1–S71, doi:https://doi.org/10.1016/j.fct.2008.06.085.
75. Peixoto, M.G.; Blank, A.F.; Arrigoni-blank, M.D.F.; GAGLIARDI, P.R.; MELO, J.O. de; NIZIO, D.A. de C.; PINTO, V.S. ACTIVITY OF ESSENTIAL OILS OF Lippia Alba CHEMOTYPES AND THEIR MAJOR MONOTERPENES AGAINST PHYTOPATHOGENIC FUNGI. *Biosci. J.* **2018**, *34*, 1136–1146.
76. Kroona, L.; Ahlgren, C.; Dahlin, J.; Isaksson, M.; Bruze, M. Use Test with L-Carvone in Toothpaste on Sensitized Individuals. *Contact Dermatitis* **2023**, *88*, 463–471, doi:10.1111/cod.14302.
77. Gimenes, L.; Silva, J.C.R.L.; Facanali, R.; Hantao, L.W.; Siqueira, W.J.; Marques, M.O.M. Essential Oils of New Lippia Alba Genotypes Analyzed by Flow-Modulated Comprehensive Two-Dimensional Gas Chromatography (GC×GC) and Chemometric Analysis. *Molecules* **2021**, *26*, doi:10.3390/molecules26082332.
78. Hatano, V.Y.; Torricelli, A.S.; Giassi, A.C.C.; Coslope, L.A.; Viana, M.B. Anxiolytic Effects of Repeated Treatment with an Essential Oil from Lippia Alba and (R)-(-)-Carvone in the Elevated T-Maze. *Brazilian J. Med. Biol. Res.* **2012**, *45*, 238–243, doi:10.1590/S0100-879X2012007500021.
79. Junior, G.B.; de Abreu, M.S.; Rosa, J.G. dos S. da; Pinheiro, C.G.; Heinzmann, B.M.; Caron, B.O.; Baldisserotto, B.; Barcellos, L.J.G. Lippia Alba and Aloysia Triphylla Essential Oils Are Anxiolytic without Inducing Aversiveness in Fish. *Aquaculture* **2018**, *482*, 49–56, doi:https://doi.org/10.1016/j.aquaculture.2017.09.023.
80. Rašković, A.; Milanović, I.; Pavlović, N.; Ćebović, T.; Vukmirović, S.; Mikov, M. Antioxidant Activity of Rosemary (Rosmarinus Officinalis L.) Essential Oil and Its Hepatoprotective Potential. *BMC Complement. Altern. Med.* **2014**, *14*, 225, doi:10.1186/1472-6882-14-225.
81. Satyal, P.; Jones, T.H.; Lopez, E.M.; McFeeters, R.L.; Ali, N.A.A.; Mansi, I.; Al-Kaf, A.G.; Setzer, W.N. Chemotypic Characterization and Biological Activity of Rosmarinus Officinalis. *Foods (Basel, Switzerland)* **2017**, *6*, doi:10.3390/foods6030020.
82. Borges, R.S.; Ortiz, B.L.S.; Pereira, A.C.M.; Keita, H.; Carvalho, J.C.T. Rosmarinus Officinalis Essential Oil: A Review of Its Phytochemistry, Anti-Inflammatory Activity, and Mechanisms of Action Involved. *J. Ethnopharmacol.* **2019**, *229*, 29–45, doi:https://doi.org/10.1016/j.jep.2018.09.038.
83. Melero-Bravo, E.; Ortiz de Elguea-Culebras, G.; Sánchez-Vioque, R.; Fernández-Sestelo, M.; Herraiz-Peñalver, D.; Sánchez-Vioque, R. Variability of Essential Oil in Cultivated Populations of Rosmarinus Officinalis L. in Spain. *Euphytica* **2022**, *218*, 65, doi:10.1007/s10681-022-03020-0.
84. Alvarado-García, P.A.A.; Soto-Vásquez, M.R.; Rosales-Cerquin, L.E.; Benites, S.M.; Cubas-Romero, T.L.; Jara-Aguilar, D.R.; Gavidia-Valencia, J.G.; Alfaro-Beltrán, I.M. Effect of Rosmarinus Officinalis Essential Oil On Anxiety, Depression, And Sleep Quality. *Pharmacogn. J.* **2023**, *15*.
85. Capatina, L.; Boiangiu, R.S.; Dumitru, G.; Napoli, E.M.; Ruberto, G.; Hritcu, L.; Todirascu-ciornea, E. Rosmarinus o Ffi Cinalis Essential Oil Improves Scopolamine-Induced Neurobehavioral Changes via Restoration of Cholinergic Function and Brain Antioxidant Status in Zebrafish (Danio Rerio). *Antioxidants* **2020**, *9*, 62, doi:10.3390/antiox9010062.
86. Galdino, P.M.; Nascimento, M.V.M.; Florentino, I.F.; Lino, R.C.; Fajemiroye, J.O.; Chaibub, B.A.; de Paula, J.R.; de Lima, T.C.M.; Costa, E.A. The Anxiolytic-like Effect of an Essential Oil Derived from Spiranthera Odoratissima A. St. Hil. Leaves and Its Major Component, β-Caryophyllene, in Male Mice. *Prog. Neuropsychopharmacol. Biol. Psychiatry* **2012**, *38*, 276–284, doi:10.1016/j.pnpbp.2012.04.012.

87. Rabbani, M.; Sajjadi, S.E.; Vaezi, A. Evaluation of Anxiolytic and Sedative Effect of Essential Oil and Hydroalcoholic Extract of Ocimum Basilicum L. and Chemical Composition of Its Essential Oil. *Res. Pharm. Sci.* **2015**, *10*.
88. Gradinariu, V.; Cioanca, O.; Hritcu, L.; Trifan, A.; Gille, E.; Hancianu, M. Comparative Efficacy of Ocimum Sanctum L. and Ocimum Basilicum L. Essential Oils against Amyloid Beta (1–42)-Induced Anxiety and Depression in Laboratory Rats. *Phytochem. Rev.* **2015**, *14*, 567–575, doi:10.1007/s11101-014-9389-6.
89. Ali, S.S.; Abd El Wahab, M.G.; Ayuob, N.N.; Suliaman, M. The Antidepressant-like Effect of Ocimum Basilicum in an Animal Model of Depression. *Biotech. Histochem.* **2017**, *92*, 390–401, doi:10.1080/10520295.2017.1323276.
90. Blanco, M.M.; Costa, C.A.R.A.; Freire, A.O.; Santos, J.G.; Costa, M. Neurobehavioral Effect of Essential Oil of Cymbopogon Citratus in Mice. *Phytomedicine* **2009**, *16*, 265–270, doi:https://doi.org/10.1016/j.phymed.2007.04.007.
91. Costa, C.A.R. de A.; Kohn, D.O.; de Lima, V.M.; Gargano, A.C.; Flório, J.C.; Costa, M. The GABAergic System Contributes to the Anxiolytic-like Effect of Essential Oil from Cymbopogon Citratus (Lemongrass). *J. Ethnopharmacol.* **2011**, *137*, 828–836, doi:https://doi.org/10.1016/j.jep.2011.07.003.
92. Cortes-Torres, A.G.; López-Castillo, G.N.; Marín-Torres, J.L.; Portillo-Reyes, R.; Luna, F.; Baca, B.E.; Sandoval-Ramírez, J.; Carrasco-Carballo, A. Cymbopogon Citratus Essential Oil: Extraction , GC – MS , Phytochemical Analysis , Antioxidant Activity , and In Silico Molecular Docking for Protein Targets Related to CNS. *Curr. Issues Mol. Biol.* **2023**, *45*, 5164–5179.
93. Alvarado-Garcia, P.A.A.; Soto-Vásquez, M.R.; Rosales-Cerquin, L.E.; Benites, S.M.; Cubas-Romero, T.L.; Ram\'\irez-Roca, E.G. Anxiolytic-Like Effect of Cymbopogon Citratus (Lemongrass) Essential Oil. *Pharmacogn. J.* **2023**, *15*, 674–679, doi:10.5530/pj.2023.15.136.
94. Goes, T.C.; Ursulino, F.R.C.; Almeida-Souza, T.H.; Alves, P.B.; Teixeira-Silva, F. Effect of Lemongrass Aroma on Experimental Anxiety in Humans. *J. Altern. Complement. Med.* **2015**, *21*, 766–773, doi:10.1089/acm.2015.0099.
94. El Azab, E.F.; Abdulmalek, S. Amelioration of Age-Related Multiple Neuronal Impairments and Inflammation in High-Fat Diet-Fed Rats: The Prospective Multitargets of Geraniol. *Oxid Med Cell Longev.* 2022, 4812993.5
